# Association Between Fat Mass or Fat Fibrotic Gene Expression and Polyneuropathy in Subjects With Obesity: A Korean Metabolic Bariatric Surgery Cohort

**DOI:** 10.3389/fendo.2022.881093

**Published:** 2022-05-16

**Authors:** Kyuho Kim, Tae Jung Oh, Young Suk Park, Won Chang, Hyen Chung Cho, Jihye Lee, Yun Kyung Lee, Sung Hee Choi, Hak Chul Jang

**Affiliations:** ^1^ Department of Internal Medicine, Seoul National University Bundang Hospital, Seongnam, South Korea; ^2^ Department of Internal Medicine, Seoul National University College of Medicine, Seoul, South Korea; ^3^ Department of Surgery, Seoul National University Bundang Hospital, Seongnam, South Korea; ^4^ Department of Radiology, Seoul National University Bundang Hospital, Seongnam, South Korea

**Keywords:** fibrosis, insulin resistance, obesity, polyneuropathy, bariatric surgery

## Abstract

**Aim:**

We aimed to investigate the association between obesity-related parameters and polyneuropathy (PN) and to evaluate inflammatory and fibrotic gene expression of fat as a potential mediator in subjects scheduled to undergo metabolic bariatric surgery (MBS).

**Methods:**

This was a cross-sectional study of MBS cohort. Body composition and visceral fat area (VFA) were quantified by bioimpedance analysis and computed tomography scan. PN was defined by Michigan Neuropathy Screening Instrument–Physical Examination score was > 2. We measured mRNA expression level of *FN1*, *TIMP1*, *CCL2*, and *CXCL8* in omental fat tissue.

**Results:**

Of 189 subjects (mean age, 39.4 years; 69 [36.5%] male; mean body mass index, 38.5 kg/m^2^), prevalence of PN was 9.1% in subjects without diabetes (n = 110) and 20.3% in those with diabetes (n = 79). Nondiabetic subjects with PN had higher homeostatic model assessment-insulin resistance (6.8 ± 3.5 *vs* 4.5 ± 2.8, *p* = 0.041), and increased fat mass (58.5 ± 12.5 kg *vs* 50.5 ± 10.7 kg, *p* = 0.034), and VFA (309.4 ± 117.6 cm^2^ *vs* 243.5 ± 94.2 cm^2^, *p* = 0.046) compared to those without PN. These obesity-related parameters were significantly associated with the presence of PN after adjusting for conventional risk factors of PN only in subjects without diabetes. In contrast, a fibrotic gene such as *TIMP1* was independently associated with PN (adjusted odds ratio of 1.56; 95% confidence interval 1.06, 2.30) only in subjects with diabetes.

**Conclusion:**

Increased adiposity was independently associated with PN in obese subjects without diabetes. In contrast, this association was not significant after adjusting conventional risk factors of PN in obese subjects with diabetes but increased fibrotic gene expression in fat was associated with PN in this group.

## Introduction

Polyneuropathy (PN), one of the most common types of peripheral neuropathy, is prevalent in subjects with diabetes and even in those with prediabetes. While increased duration of diabetes and poor glycemic control are established risk factors for diabetic PN (1, 2), intensive glycemic control shows limited efficacy in prevention of PN in type 2 diabetes (3). This suggests that risk factors other than glucose, such as the components of metabolic syndrome, might contribute to nerve damage to a considerable extent.

The Cooperative Health Research in the Region of Augsburg (KORA) study (4) and the Anglo–Danish–Dutch study of Intensive Treatment in People with Screen-Detected Diabetes in Primary Care (ADDITION) study (5) revealed an association between general and abdominal obesity (weight and waist circumference) and development of PN. Besides the amount of fat, the quality of the fat tissue could induce detrimental effects on the metabolic milieu. Considering that chronic adipose tissue inflammation was associated with obesity-related metabolic complications (6) and adipose tissue is the main site where systemic inflammation begins (7), it is logically reasonable to investigate an association of the expression level of inflammatory and fibrosis genes in fat tissue with PN.

Meanwhile, a previous observational study showed that 11.1% of obese subjects already had PN despite normoglycemia, and that the presence of PN is positively associated with waist circumference (8). This suggests that the association between obesity and PN needs to be analyzed in a subgroup stratified by diabetes status.

Therefore, in this study, we aimed to investigate the association between obesity-related parameters and PN in obese subjects stratified by diabetes status. We used computed tomography scan to assess the quantity of VAT, and analyzed mRNA levels of inflammatory and fibrosis genes such as *FN1* (9), *TIMP1* (10), *CCL2* (11), and *CXCL8* (12) from omental fat tissue. These genes have been studies as a driver of systemic inflammation and insulin resistance.

## Methods

### Population

**Figure 1:**
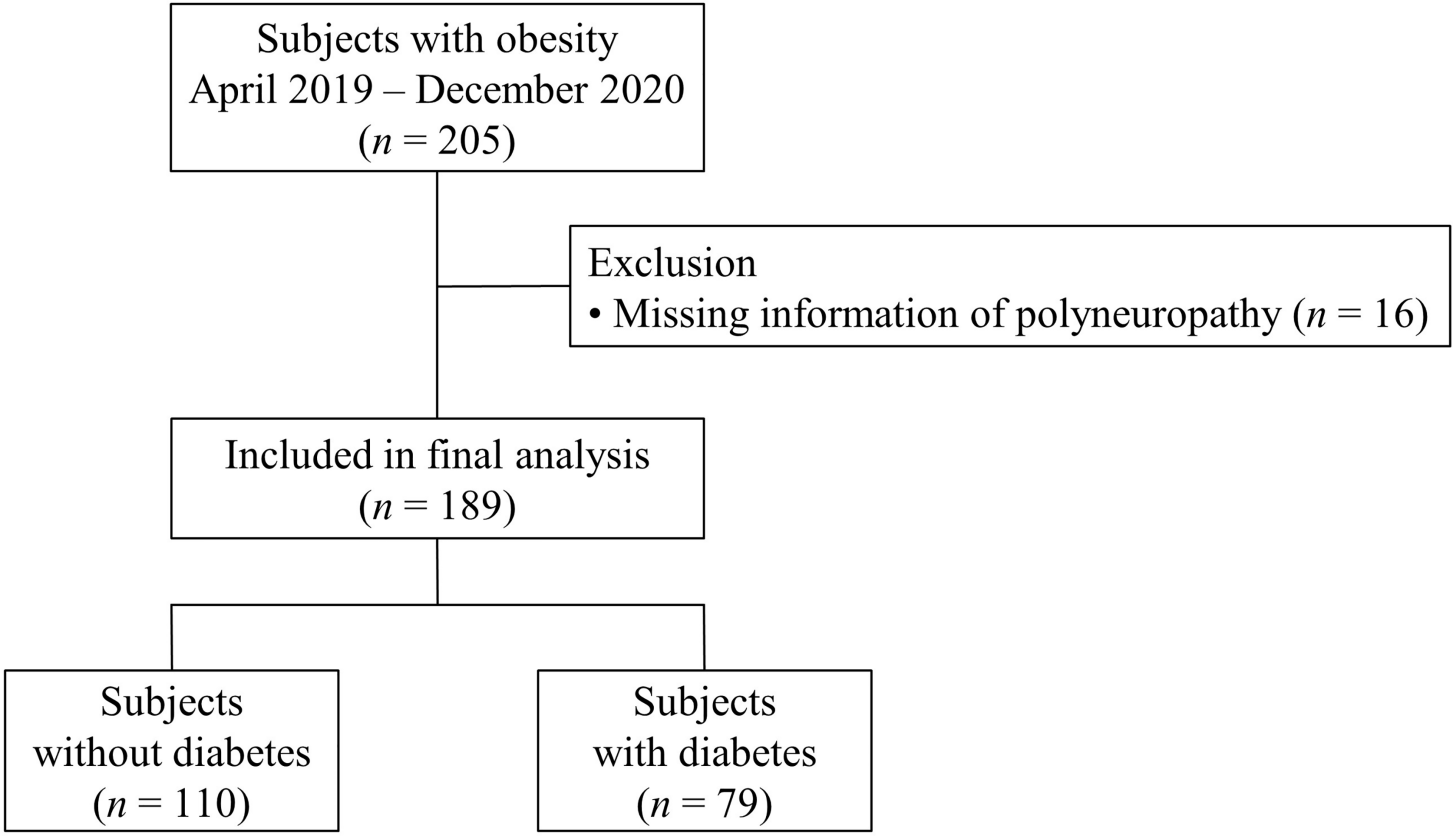
Flow chart of the selection of subjects for the analysis. Among 205 obese subjects, after excluding subjects with missing information of polyneuropathy, 189 subjects were included in the final analysis. They were divided into two groups according to diabetes status.

We recruited subjects scheduled to undergo metabolic bariatric surgery (MBS) and intensively evaluated metabolic parameters and the presence of diabetic vascular complications. A total of 205 obese subjects were enrolled from Seoul National University Bundang Hospital (SNUBH), a tertiary academic hospital from April 2019 to December 2020, the aim of original prospective observational study is to discover predictive markers for weight loss and metabolic improvement after MBS. The original cohort study has been registered at Clinical research Information Service (CRIS Registration No. KCT0005777). Inclusion criteria were age ≥ 20 years old and a body mass index (BMI) ≥ 35 kg/m^2^ with no comorbidity; BMI ≥ 30 kg/m^2^ with at least one comorbidity; or BMI ≥ 27.5 kg/m^2^ with medically uncontrolled type 2 diabetes. Type 2 diabetes was defined according to the criteria of American Diabetes Association: fasting plasma glucose (FPG) ≥ 7.0 mmol/l or HbA_1c_ ≥ 6.5% (48 mmol/mol) (13). Subjects on diabetes medications were also considered to have diabetes. We excluded the subjects who did not meet the indication of MBS according to the local guideline (14). We also excluded the subjects who previously underwent MBS. This study was a cross-sectional study analyzing data of 189 participants (**Figure 1**) after excluding subjects with missing information of PN (*n* = 16). We applied 2:1 propensity score matching using age, sex, and BMI to compare mRNA expression levels in omental fat tissue between subjects without PN and those with PN. However, values were excluded in analysis if quality of cDNA sample was unacceptable. The study was approved by the Institutional Review Board of SNUBH (no. B-2111-718-301), and each participant provided written informed consent. The study was conducted in accordance with the Declaration of Helsinki.

### Anthropometric and Biochemical Analyses

Anthropometric indices were measured by a well-trained research nurse. BMI was calculated as weight (kg) divided by the square of the height in meters. Waist circumference was measured at the midpoint between the margin of the lowest rib and the iliac crest. Systolic blood pressure (BP) and diastolic BP were measured by an electronic BP monitor after 10 minutes of rest in a sitting position. Smoking status was classified as never smoker (< 100 cigarettes in a lifetime and currently a nonsmoker), ex-smoker (≥ 100 cigarettes in a lifetime and currently a nonsmoker), and current smoker (≥ 100 cigarettes in a lifetime and currently a smoker). We defined drinkers as those who drank any alcoholic beverage more than once a month. Positive exercise was defined as exercising for > 150 min/week. Blood samples were collected after an overnight fast. FPG levels were measured by the hexokinase method, and glycated hemoglobin (HbA_1c_) levels were measured by high-performance liquid chromatography (Bio-Rad, Hercules, CA, USA). Serum insulin levels were measured by immunoradiometric assay (DIAsource, Nivelles, Belgium). Total cholesterol, triglyceride, high-density lipoprotein (HDL) cholesterol, and low-density lipoprotein (LDL) cholesterol were measured by enzymatic colorimetric assay. Creatinine was measured by the protocol of the central laboratory of SNUBH, and estimated glomerular filtration rate (eGFR) was calculated by the Modification of Diet in Renal Disease equation (15). Homeostasis model assessment-insulin resistance (HOMA-IR) was calculated using the following formula: HOMA-IR = (fasting insulin [μU/ml] × FPG [mmol/l]/22.5) (16).

### Body Composition Measurements

Body fat mass, fat percent, and lean body mass (LBM) were estimated by a bioimpedance analysis (InBody770, InBody, Seoul, Korea). Regarding the visceral fat area (VFA), cross-sectional abdominal computed tomography (CT) images at the level of the third lumbar vertebral body (L3) were acquired, and VFA was calculated from areas within a range of –150 to –50 Hounsfield units (17).

### Assessment of PN

We used the Michigan Neuropathy Screening Instrument (MNSI), which includes two separate assessments, a 15-item self-administered questionnaire (MNSI-Q) and a lower-extremity physical examination (MNSI-PE) (18). PN was diagnosed when the MNSI-PE score was > 2. A trained nurse performed all neurologic examinations.

### Measurement of Inflammatory and Fibrosis Markers

Omental fat tissue was obtained during MBS and stored at –80°C. Total RNA was extracted from frozen human fat tissue samples using TRIzol (Thermo Fisher Scientific, Waltham, MA, USA). For quantitative real-time PCR analysis, 1 μg of total RNA was reverse-transcribed using the High-Capacity cDNA Reverse Transcription kit (Thermo Fisher Scientific, Waltham, MA, USA). SYBR Green reactions using the SYBR Green PCR Master mix (Enzynomics, Daejeon, Korea) were assembled along with primers according to the manufacturer’s instructions and were performed using the QuantStudio 7 Flex Real-Time PCR System (Thermo Fisher Scientific, Waltham, MA, USA). Relative mRNA levels were calculated using the comparative threshold cycle method and normalized to *cyclophilin* mRNA. All primers used are listed with their sequences in **Supplementary Table 1**.

### Statistical Analysis

Data were expressed as the mean ± standard deviation or number (%). For checking normality of distribution of variables, Kolmogorov-Smirnov and Shapiro-Wilk tests were used. Categorical variables were compared using χ^2^ tests, and continuous variables were compared using Student’s unpaired *t* tests for parametric data or Mann–Whitney U tests for nonparametric data. Since variables were not normally distributed, Spearman’s correlation coefficient was used to evaluate the correlation between variables. Univariable and multivariable logistic regression models were used to analyze the associations between obesity-related parameters and PN. In all cases, *p* < 0.05 was considered statistically significant. Statistical analyses were performed using IBM SPSS version 25.0 (IBM Inc., Armonk, NY, USA). Figures were drawn using GraphPad Prism software (version 9.1.2; GraphPad Software Inc., CA, USA).

## Results

Among 189 subjects who were candidates for MBS, 79 (41.8%) had diabetes. Prevalence of PN was 9.1% in subjects without diabetes and 20.3% in those with diabetes. Among subjects without diabetes, insulin and HOMA-IR were higher in subjects with PN compared with those without PN. Among subjects with diabetes, body weight, BMI, waist circumference, eGFR, and the proportion of subjects taking insulin therapy were higher in subjects with PN compared with those without PN. However, diabetes duration, FPG, and HbA_1c_ were comparable between subjects with PN and those without PN (**Table 1**). Body composition analysis showed that fat mass and VFA were higher in subjects with PN compared with those without PN, irrespective of diabetes status. Among subjects with diabetes, LBM was higher in subjects with PN compared with those without PN (**Table 1**).

**Table 1:** Baseline characteristics of obese subjects stratified by diabetes and polyneuropathy.

Variable	Diabetes (–) (*n* = 110)		*p* value	Diabetes (+) (*n* = 79)		*p* value
	PN (–) (*n* = 100)	PN (+) (*n* = 10)		PN (–) (*n* = 63)	PN (+) (*n* = 16)	
Male, *n* (%)	34 (34.0)	3 (30.0)	1.000	24 (38.1)	8 (50.0)	0.386
Age (years)	36.5 ± 9.3	38.3 ± 15.9	0.983	42.6 ± 10.6	45.1 ± 12.7	0.490
Body weight (kg)	107.8 ± 20.2	121.2 ± 28.4	0.154	101.6 ± 23.5	118.7 ± 22.7	0.003
BMI (kg/m^2^)	38.7 ± 5.2	42.1 ± 7.7	0.206	36.8 ± 5.9	42.3 ± 7.5	0.005
Obesity classes, *n* (%)			0.496			0.144
Class I (BMI 25–29.9)	1 (1.0)	0 (0.0)		5 (7.9)	0 (0.0)	
Class II (BMI 30–34.9)	26 (26.0)	1 (10.0)		23 (36.5)	3 (18.8)	
Class III (BMI ≥ 35)	73 (73.0)	9 (90.0)		35 (55.6)	13 (81.3)	
Waist circumference (cm)	115.2 ± 12.3	117.8 ± 11.0	0.571	113.2 ± 14.4	124.7 ± 15.9	0.007
Systolic BP (mmHg)	134.0 ± 15.8	134.2 ± 13.6	0.743	136.3 ± 19.0	145.1 ± 15.7	0.089
Diastolic BP (mmHg)	80.4 ± 12.0	78.3 ± 10.8	0.685	83.0 ± 13.4	80.3 ± 15.2	0.526
Diabetes duration (years)	NA	NA	NA	4.5 ± 5.3	10.0 ± 10.8	0.074
FPG (mmol/l)	5.5 ± 0.6	5.5 ± 0.5	0.720	8.5 ± 3.1	8.9 ± 3.0	0.522
HbA_1c_ (%)	5.5 ± 0.4	5.6 ± 0.7	0.707	7.8 ± 1.8	7.3 ± 1.3	0.490
HbA_1c_ (mmol/mol)	36.5 ± 4.0	37.1 ± 7.7	0.707	61.7 ± 19.5	56.4 ± 14.4	0.490
Triglyceride (mmol/l)	1.8 ± 1.5	1.9 ± 0.9	0.491	2.1 ± 1.4	2.1 ± 0.9	0.357
HDL cholesterol (mmol/l)	1.4 ± 0.3	1.2 ± 0.2	0.091	1.3 ± 0.2	1.3 ± 0.2	0.877
LDL cholesterol (mmol/l)	3.4 ± 0.6	3.2 ± 0.4	0.574	2.9 ± 0.9	3.0 ± 0.7	0.353
eGFR (mL min^-1^ [1.73 m]^2^)	115.6 ± 24.0	124.5 ± 34.6	0.743	110.8 ± 24.8	94.7 ± 28.6	0.025
Insulin (pmol/l)	126.5 ± 72.8	190.2 ± 94.7	0.042	126.0 ± 91.8	136.0 ± 100.6	0.829
HOMA-IR	4.5 ± 2.8	6.8 ± 3.5	0.041	6.6 ± 4.4	7.0 ± 4.7	0.830
10-g monofilament, Right	9.8 ± 0.5	9.4 ± 0.7	0.013	9.6 ± 0.6	9.3 ± 0.9	0.148
10-g monofilament, Left	9.8 ± 0.5	9.3 ± 0.8	0.012	9.7 ± 0.6	9.4 ± 0.7	0.198
MNSI-Q	2.2 ± 1.8	3.3 ± 2.1	0.076	3.2 ± 2.1	4.6 ± 2.0	0.019
MNSI-PE	0.4 ± 0.7	3.3 ± 0.5	<0.001	0.6 ± 0.7	3.2 ± 0.5	<0.001
Smoking status			0.768			0.201
Never smoker, *n* (%)	67 (67.0)	6 (60.0)		27 (42.9)	10 (62.5)	
Ex-smoker, *n* (%)	12 (12.0)	2 (20.0)		14 (22.2)	4 (25.0)	
Current smoker, *n* (%)	21 (21.0)	2 (20.0)		22 (34.9)	2 (12.5)	
Alcohol, *n* (%)	74 (74.0)	7 (70.0)	0.784	38 (60.3)	10 (62.5)	0.873
Exercise, *n* (%)	43 (43.0)	4 (40.0)	0.834	30 (47.6)	3 (18.8)	0.048
Hypertension, *n* (%)	36 (36.0)	5 (50.0)	0.383	39 (61.9)	12 (75.0)	0.328
Dyslipidaemia, *n* (%)	37 (37.0)	4 (40.0)	0.852	45 (71.4)	10 (62.5)	0.488
Insulin therapy, *n* (%)	NA	NA	NA	10 (15.9)	7 (43.8)	0.015
Fat mass (kg)	50.5 ± 10.7	58.4 ± 12.5	0.034	44.2 ± 13.8	54.0 ± 16.4	0.028
Fat percent (%)	46.1 ± 6.5	48.5 ± 4.0	0.165	43.2 ± 6.3	44.9 ± 7.6	0.390
LBM (kg)	58.0 ± 12.8	62.9 ± 17.1	0.431	57.1 ± 13.1	64.6 ± 12.2	0.012
VFA (cm^2^)	243.5 ± 94.2	309.4 ± 117.6	0.046	271.7 ± 78.7	334.2 ± 113.4	0.020

Data are expressed as the mean ± standard deviation or number (%).

BMI, body mass index; BP, blood pressure; eGFR, estimated glomerular filtration rate; FPG, fasting plasma glucose; HbA_1c_, glycated haemoglobin; HDL, high-density lipoprotein; HOMA-IR, homeostasis model assessment-insulin resistance; LBM, lean body mass; LDL, low-density lipoprotein; MNSI-PE, Michigan Neuropathy Screening Instrument-Physical Examination; MNSI-Q, Michigan Neuropathy Screening Instrument-Questionnaire; NA, not applicable; PN, polyneuropathy; VFA, visceral fat area.

Among subjects without diabetes, unadjusted odds ratio (OR) (95% confidence interval [CI]) for PN was 1.23 (1.02, 1.49) for HOMA-IR and 1.06 (1.00, 1.12) for fat mass. Among subjects with diabetes, unadjusted OR (95% CI) for PN was 1.05 (1.01, 1.09) for waist circumference, 1.13 (1.03, 1.23) for BMI, 1.04 (1.01, 1.08) for fat mass, and 1.01 (1.00, 1.02) for VFA. After adjustment of known risk factors for PN (19), adjusted OR (95% CI) for PN was 1.47 (1.10, 1.95) for HOMA-IR, 1.08 (1.00, 1.16) for fat mass, and 1.01 (1.00, 1.02) for VFA in subjects without diabetes. In contrast, waist circumference, BMI, fat mass, and VFA showed no significant association with PN in subjects with diabetes after adjustment of covariates (**Table 2**).

**Table 2:** ORs and 95% CIs for polyneuropathy stratified by diabetes.

Variable	Diabetes (-) (*n* = 110)				Diabetes (+) (*n* = 79)			
	Unadjusted OR (95% CI)	*p* value	Adjusted OR (95% CI)	*p* value	Unadjusted OR (95% CI)	*p* value	Adjusted OR (95% CI)	*p* value
Age	1.02 (0.96–1.09)	0.585	–	–	1.02 (0.97–1.07)	0.415	–	–
Sex								
Female	1.20 (0.29–4.95)	0.799	–	–	0.62 (0.20–1.86)	0.389	–	–
Male (Reference)	1	1	–	–	1	1	–	–
BMI	1.11 (0.99–1.23)	0.067	1.12 (0.98–1.28)	0.095	1.13 (1.03–1.23)	0.006	1.12 (0.99–1.27)	0.066
Waist circumference	1.02 (0.96–1.08)	0.547	1.04 (0.96–1.12)	0.372	1.05 (1.01–1.09)	0.014	1.04 (0.98–1.09)	0.180
Systolic BP	1.00 (0.96–1.04)	0.966	–	–	1.03 (1.00–1.06)	0.096	–	–
Diabetes duration	–	–	–	–	1.01 (1.02–1.18)	0.012	–	–
HbA_1c_	1.49 (0.29–7.59)	0.634	–	–	0.82 (0.56–1.20)	0.303	–	–
Triglyceride	1.00 (1.00–1.01)	0.878	–	–	1.00 (1.00–1.01)	0.937	–	–
HDL cholesterol	0.95 (0.88–1.01)	0.114	–	–	1.00 (0.94–1.06)	0.917	–	–
HOMA-IR	1.23 (1.02–1.49)	0.031	1.47 (1.10–1.95)	0.009	1.02 (0.90–1.16)	0.751	1.06 (0.90–1.25)	0.521
Smoking status								
Current smoker	0.94 (0.19–4.76)	0.941	–	–	0.27 (0.06–1.28)	0.098	–	–
Ex-smoker	1.86 (0.33–10.33)	0.478	–	–	0.77 (0.21–2.91)	0.702	–	–
Never smoker (Reference)	1	1	–	–	1	1	–	–
Alcohol status								
Drinker	0.82 (0.20–3.41)	0.785	–	–	1.10 (0.35–3.40)	0.873	–	–
Non-drinker (Reference)	1	1	–	–	1	1	–	–
Exercise	0.87 (0.23–3.27)	0.835	–	–	0.25 (0.07–0.98)	0.046	–	–
Fat mass	1.06 (1.00–1.12)	0.040	1.08 (1.01–1.16)	0.034	1.04 (1.01–1.08)	0.025	1.04 (0.99–1.10)	0.137
VFA	1.01 (1.00–1.01)	0.052	1.01 (1.00–1.02)	0.031	1.01 (1.00–1.02)	0.021	1.00 (1.00–1.01)	0.440
*TIMP1* ^a^	1.13 (0.82–1.57)	0.459	1.41 (0.77–2.56)	0.262	1.34 (1.09–1.66)	0.007	1.51 (1.02–2.23)	0.040
*CXCL8* ^b^	0.97 (0.85–1.11)	0.705	1.14 (0.88–1.47)	0.317	1.68 (1.04–2.70)	0.033	1.90 (0.92–3.92)	0.081

Data are presented as ORs and 95% CIs.

Adjusted analysis: adjustment for age, sex, systolic blood pressure, glycated haemoglobin (HbA_1c_), triglyceride, HDL cholesterol, smoking status, alcohol status, and exercise (+ diabetes duration in diabetes (+) group). In diabetes (+) group, for TIMP1 and CXCL8, VFA was adjusted additionally.

a Diabetes (–) (n = 26), Diabetes (+) (n = 47).

b Diabetes (–) (n = 27), Diabetes (+) (n = 46).

BMI, body mass index; BP, blood pressure; CI, confidence interval; HDL, high-density lipoprotein; HOMA-IR, homeostasis model assessment-insulin resistance; OR, odds ratio; VFA, visceral fat area.

**Figure 2:**
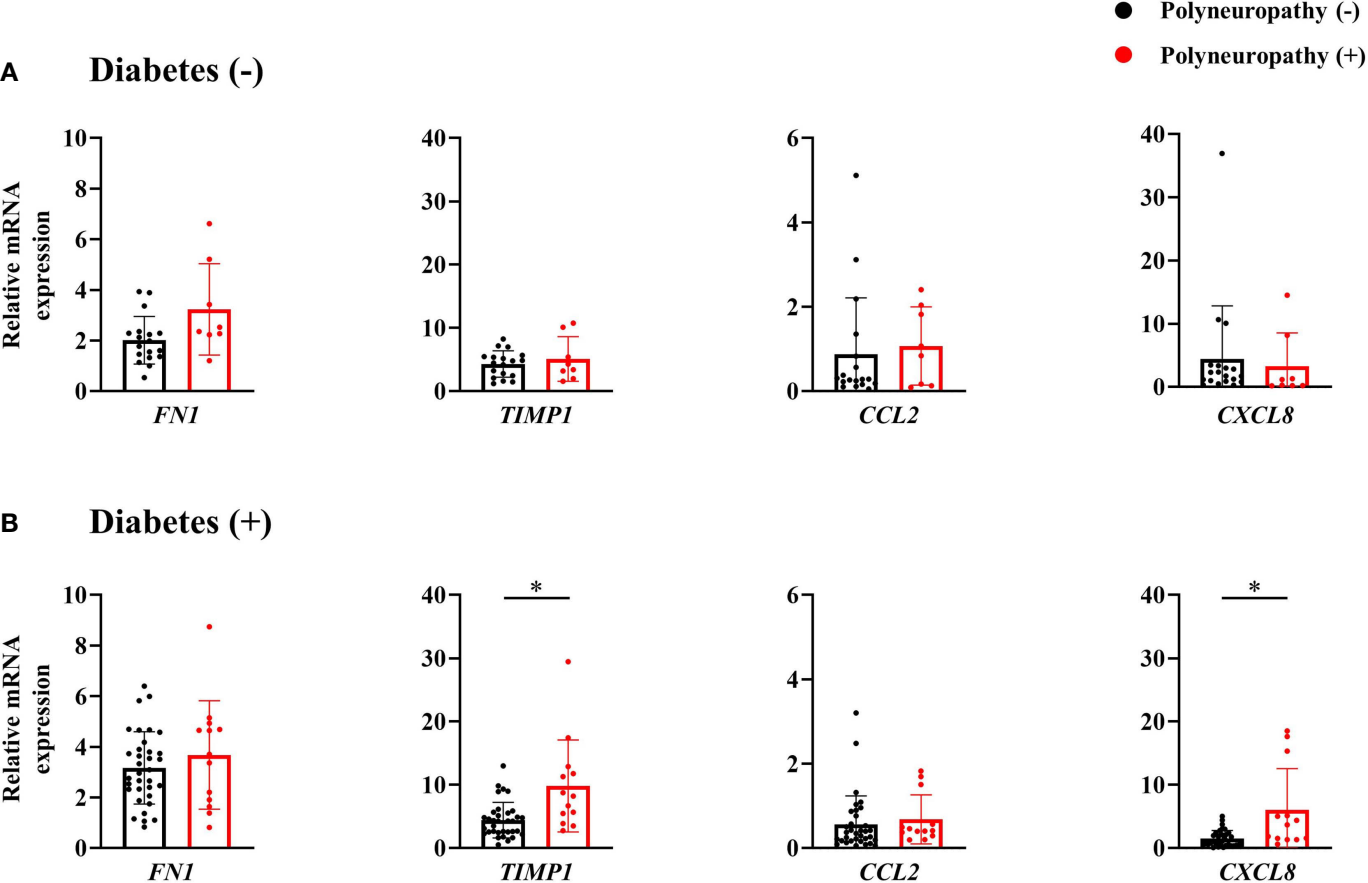
mRNA expression of inflammatory and fibrotic genes of omental fat tissue in subjects without diabetes **(A)** and those with diabetes **(B)**. ^*^ *p* < 0.05.

**Figure 3:**
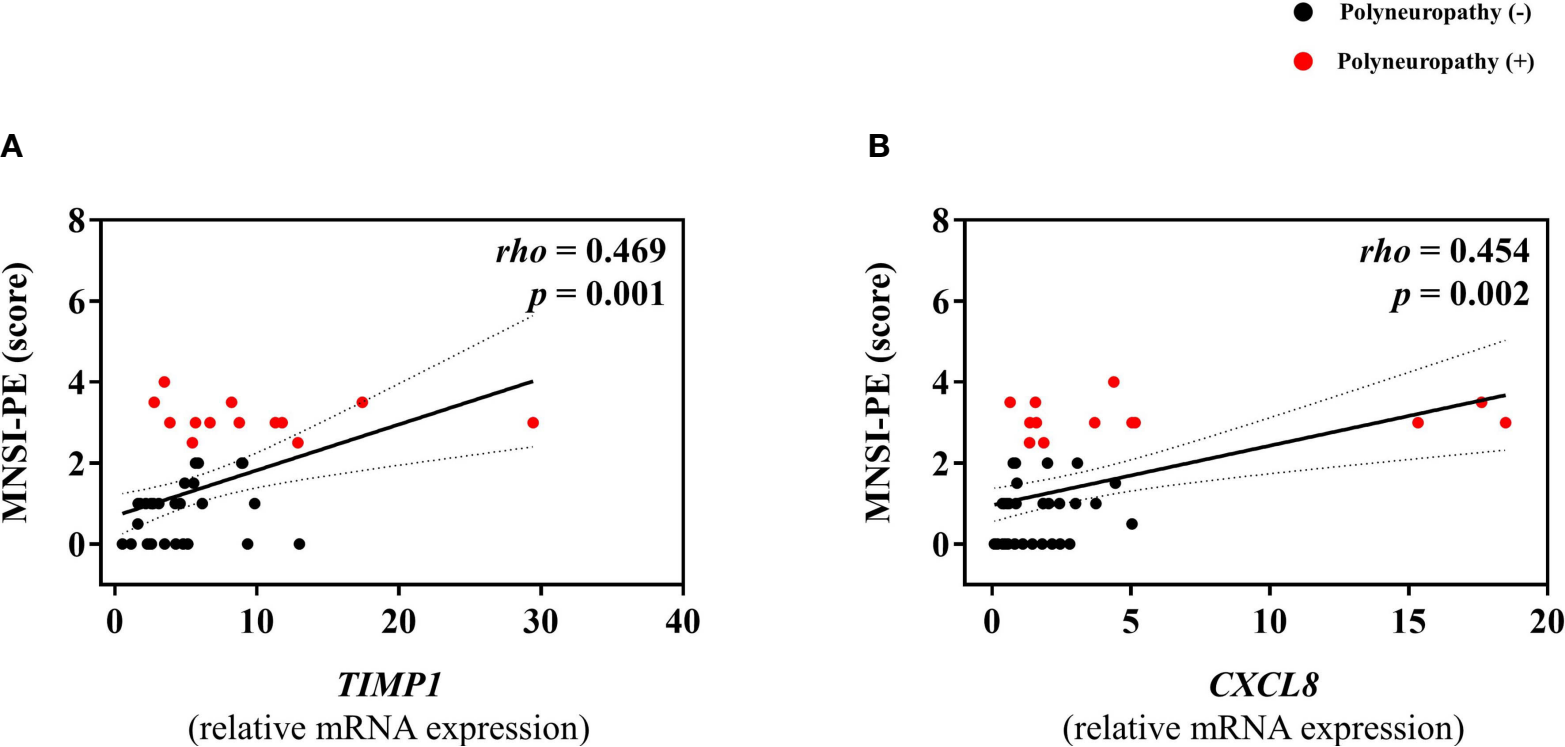
Correlations between mRNA expression level of *TIMP1* **(A)** or *CXCL8* **(B)**, and MNSI-PE scores in subjects with diabetes.

Among subjects without diabetes, any mRNA expression of omental fat was not different according to the presence of PN. In contrast, among subjects with diabetes, mRNA expression of *TIMP1* and *CXCL8* was significantly higher in subjects with PN (**Figure 2**), and positively correlated with MNSI-PE scores (*rho* = 0.469, *p* = 0.001; *rho* = 0.454, *p* = 0.002) (**Figure 3**). In addition, adjusted OR (95% CI) for PN was 1.56 (1.06, 2.30) for *TIMP1* in subjects with diabetes (**Table 2**).

## Discussion

In this study, we found increased fat mass and VFA, and higher HOMA-IR in subjects with PN compared with those without PN among subjects without diabetes. These variables were still significantly associated with PN even after adjusting for known risk factors for PN in this group. On the other hand, among subjects with diabetes we found no difference in HOMA-IR between subjects with or without PN, and the association of fat mass and VFA with PN was not significant after adjusting covariates. In contrast to these insignificant associations, *TIMP1* gene expression as a marker of fat fibrosis was significantly higher in the presence of PN in the diabetes subgroup.

Hyperglycemia is a major risk factor for PN (19), and glucose control is the mainstay of prevention and management of PN (20). However, enhanced glucose control modestly reduced (21), or did not reduce the incidence of PN in type 2 diabetes (22, 23). Therefore, it is necessary to identify other risk factors for PN in subjects with type 2 diabetes or at high risk for type 2 diabetes. From the data of obese subjects without diabetes, we confirmed that obesity is the main metabolic driver of PN. Furthermore, insulin resistance assessed by HOMA-IR was also an independent risk factor for PN in this population. Considering that insulin is a neurotrophic factor responsible for neuronal growth, survival, and differentiation (24, 25), it is possible that disruption of insulin signaling due to insulin resistance contributed to the pathogenesis of PN. However, the association between HOMA-IR and PN was not significant in subjects with overt diabetes, a finding that might be due to the strong influence of severe insulin resistance in these subjects who were candidates for MBS. Therefore, the impact of obesity on PN might depend on whether the severity of insulin resistance of subjects caused diabetes.

A prospective study of the general population with and without diabetes from the KORA cohort showed that serum levels of CCL7, CXCL10, and DNER partly mediated the association between obesity and PN (4). Another prospective study of subjects with diabetes showed an association of plasma levels of TNF and ICAM1 with development of PN (26). A cross-sectional study showed associations of plasma levels of MMP9 and TIMP1 with PN in type 1 diabetes (27). Considering that adipose tissue is the main site where systemic inflammation begins (7), it is reasonable to investigate an association of the expression level of inflammatory and fibrosis genes in fat tissue with PN. In our analysis, including subjects with diabetes, mRNA levels of *TIMP1* and *CXCL8* were significantly higher in subjects with PN compared with their counterparts. These results suggest the possibility that inflammatory cytokines released from VAT during tissue inflammation and fibrosis contribute to the pathogenesis of PN in diabetes. In contrast, no significant association between inflammatory gene expression in the omental fat and PN was found in the nondiabetic subgroup. Therefore, inflammation or fibrosis of fat tissue might contribute to PN differently according to diabetes status, however this hypothesis needs to be investigated in further study.

The current study has a number of strengths. First, we performed analysis of PN stratified by diabetes status, thereby suggesting the step-wise contribution of hyperglycemia and obesity involved in the pathogenesis of PN. Second, VFA was measured by CT scan, the gold standard method. Finally, we directly measured gene expression from omental fat tissue. To the best of our knowledge, this study is the first analysis combining gene expression of fat tissue and PN. Nevertheless, this study has some limitations. First, there were no lean controls, and we analyzed the mRNA data in the subgroup due to the availability of fat tissue. Second, a neurophysiological study was not used to confirm PN. Therefore, the diagnosis of PN is not confirmative, but possible PN was adopted. Third, we cannot establish a causal relationship based on the cross-sectional study design. Fourth, because the original cohort is based on a single center in Korea, results cannot be generalized to other races or ethnicities. Finally, we did not separate subjects with prediabetes from subjects without diabetes.

In conclusion, fat mass, VFA (“quantity of fat”), and HOMA-IR were independent risk factors for PN in obese subjects without diabetes. In contrast, advanced pathology of fat tissue such as fibrosis (“quality of fat”) might be a considerable abnormality for PN in obese subjects who already have diabetes. Therefore, the early evaluation of PN and the early management of obesity might be necessary in obese subjects at high risk of diabetes. Future studies are needed to test whether weight loss intervention can prevent and delay PN in these high-risk subjects.

## Data Availability Statement

The original contributions presented in the study are included in the article/**Supplementary Material**, further inquiries can be directed to the corresponding author.

## Ethics Statement

The studies involving human participants were reviewed and approved by the Institutional Review Board of SNUBH. The patients/participants provided their written informed consent to participate in this study.

## Author Contributions

KK drafted the article. TJO, YSP, WC, SHC, and HCJ contributed to the conception and design of the study. KK performed the statistical analyses. KK, TJO, YSP, WC, HCC, JL, and YKL contributed to the acquisition of data. TJO is the guarantor of this work. All authors critically reviewed the manuscript and approved this version to be published.

## Funding

This work was supported by the Korean Society for the Study of Obesity (Grant No. KSSO201904); the National Research Foundation of Korea (NRF) (Grant No. NRF-2020R1C1C1013766); the Medical Research Center through the NRF (Grant No. NRF-2018R1A5A2024425) funded by the Korea Government; and the SNUBH Research Fund (Grant No. 13-2019-0013).

## Conflict of Interest

The authors declare that the research was conducted in the absence of any commercial or financial relationships that could be construed as a potential conflict of interest.

## Publisher’s Note

All claims expressed in this article are solely those of the authors and do not necessarily represent those of their affiliated organizations, or those of the publisher, the editors and the reviewers. Any product that may be evaluated in this article, or claim that may be made by its manufacturer, is not guaranteed or endorsed by the publisher.

## Abbreviations

ADDITION, Anglo–Danish–Dutch study of Intensive Treatment in People with Screen-Detected Diabetes in Primary Care; BMI, Body mass index; BP, Blood pressure; CI, confidence interval; CT, Computed tomography; DNER, Delta/Notch-like epidermal growth factor-related receptor; eGFR, Estimated glomerular filtration rate; FPG, Fasting plasma glucose; HbA1_c_, Glycated hemoglobin; HDL, High-density lipoprotein; HOMA-IR, Homeostasis model assessment-insulin resistance; KORA, Cooperative Health Research in the Region of Augsburg; LBM, Lean body mass; LDL, Low-density lipoprotein; MBS, Metabolic bariatric surgery; MNSI-PE, Michigan Neuropathy Screening Instrument-Physical Examination; MNSI-Q, Michigan Neuropathy Screening Instrument-Questionnaire; OR, odds ratio; PN, Polyneuropathy; SAT, Subcutaneous adipose tissue; SNUBH, Seoul National University Bundang Hospital; VAT, Visceral adipose tissue; VFA, Visceral fat area.

## References

[1] Adler AI Boyko EJ Ahroni JH Stensel V Forsberg RC Smith DG. Risk Factors for Diabetic Peripheral Sensory Neuropathy. Results of the Seattle Prospective Diabetic Foot Study. Diabetes Care (1997) 20(7):1162–7. doi: 10.2337/diacare.20.7.1162 9203456

[2] Yang H Sloan G Ye Y Wang S Duan B Tesfaye S. New Perspective in Diabetic Neuropathy: From the Periphery to the Brain, A Call for Early Detection, and Precision Medicine. Front Endocrinol (Lausanne) (2019) 10:929. doi: 10.3389/fendo.2019.00929 32010062 PMC6978915

[3] Callaghan BC Little AA Feldman EL Hughes RA. Enhanced Glucose Control for Preventing and Treating Diabetic Neuropathy. Cochrane Database Syst Rev (2012), CD007543. doi: 10.1002/14651858.CD007543.pub2 22696371 PMC4048127

[4] Schlesinger S Herder C Kannenberg JM Huth C Carstensen-Kirberg M Rathmann W. General and Abdominal Obesity and Incident Distal Sensorimotor Polyneuropathy: Insights Into Inflammatory Biomarkers as Potential Mediators in the Kora F4/Ff4 Cohort. Diabetes Care (2019) 42(2):240–7. doi: 10.2337/dc18-1842 30523031

[5] Andersen ST Witte DR Dalsgaard EM Andersen H Nawroth P Fleming T. Risk Factors for Incident Diabetic Polyneuropathy in a Cohort With Screen-Detected Type 2 Diabetes Followed for 13 Years: Addition-Denmark. Diabetes Care (2018) 41(5):1068–75. doi: 10.2337/dc17-2062 29487078

[6] Zatterale F Longo M Naderi J Raciti GA Desiderio A Miele C. Chronic Adipose Tissue Inflammation Linking Obesity to Insulin Resistance and Type 2 Diabetes. Front Physiol (2019) 10:1607. doi: 10.3389/fphys.2019.01607 32063863 PMC7000657

[7] Xu H Barnes GT Yang Q Tan G Yang D Chou CJ. Chronic Inflammation in Fat Plays a Crucial Role in the Development of Obesity-Related Insulin Resistance. J Clin Invest (2003) 112(12):1821–30. doi: 10.1172/JCI19451 PMC296998 14679177

[8] Callaghan BC Xia R Reynolds E Banerjee M Rothberg AE Burant CF. Association Between Metabolic Syndrome Components and Polyneuropathy in an Obese Population. JAMA Neurol (2016) 73(12):1468–76. doi: 10.1001/jamaneurol.2016.3745 PMC5217829 27802497

[9] Chun TH. Peri-Adipocyte Ecm Remodeling in Obesity and Adipose Tissue Fibrosis. Adipocyte (2012) 1(2):89–95. doi: 10.4161/adip.19752 23700517 PMC3609086

[10] Meissburger B Stachorski L Roder E Rudofsky G Wolfrum C. Tissue Inhibitor of Matrix Metalloproteinase 1 (Timp1) Controls Adipogenesis in Obesity in Mice and in Humans. Diabetologia (2011) 54(6):1468–79. doi: 10.1007/s00125-011-2093-9 21437772

[11] Dahlman I Kaaman M Olsson T Tan GD Bickerton AS Wahlen K. A Unique Role of Monocyte Chemoattractant Protein 1 Among Chemokines in Adipose Tissue of Obese Subjects. J Clin Endocrinol Metab (2005) 90(10):5834–40. doi: 10.1210/jc.2005-0369 16091493

[12] Kobashi C Asamizu S Ishiki M Iwata M Usui I Yamazaki K. Inhibitory Effect of Il-8 on Insulin Action in Human Adipocytes *Via* Map Kinase Pathway. J Inflammation (Lond) (2009) 6:25. doi: 10.1186/1476-9255-6-25 PMC2746203 19709445

[13] American Diabetes A. 2. Classification and Diagnosis of Diabetes: Standards of Medical Care in Diabetes-2021. Diabetes Care (2021) 44(Suppl 1):S15–33. doi: 10.2337/dc21-S002 33298413

[14] Kim BY Kang SM Kang JH Kang SY Kim KK Kim KB. 2020 Korean Society for the Study of Obesity Guidelines for the Management of Obesity in Korea. J Obes Metab Syndr (2021) 30(2):81–92. doi: 10.7570/jomes21022 34045368 PMC8277596

[15] Levey AS Coresh J Greene T Stevens LA Zhang YL Hendriksen S. Using Standardized Serum Creatinine Values in the Modification of Diet in Renal Disease Study Equation for Estimating Glomerular Filtration Rate. Ann Intern Med (2006) 145(4):247–54. doi: 10.7326/0003-4819-145-4-200608150-00004 16908915

[16] Matthews DR Hosker JP Rudenski AS Naylor BA Treacher DF Turner RC. Homeostasis Model Assessment: Insulin Resistance and Beta-Cell Function From Fasting Plasma Glucose and Insulin Concentrations in Man. Diabetologia (1985) 28(7):412–9. doi: 10.1007/BF00280883 3899825

[17] Lee JK Park YS Kim K Oh TJ Chang W. Comparison of Bioelectrical Impedance Analysis and Computed Tomography on Body Composition Changes Including Visceral Fat After Bariatric Surgery in Asian Patients With Obesity. Obes Surg (2021) 31(10):4243–50. doi: 10.1007/s11695-021-05569-6 34283378

[18] Feldman EL Stevens MJ Thomas PK Brown MB Canal N Greene DA. A Practical Two-Step Quantitative Clinical and Electrophysiological Assessment for the Diagnosis and Staging of Diabetic Neuropathy. Diabetes Care (1994) 17(11):1281–9. doi: 10.2337/diacare.17.11.1281 7821168

[19] Papanas N Ziegler D. Risk Factors and Comorbidities in Diabetic Neuropathy: An Update 2015. Rev Diabetes Stud (2015) 12(1-2):48–62. doi: 10.1900/RDS.2015.12.48 PMC5397983 26676661

[20] Pop-Busui R Boulton AJ Feldman EL Bril V Freeman R Malik RA. Diabetic Neuropathy: A Position Statement by the American Diabetes Association. Diabetes Care (2017) 40(1):136–54. doi: 10.2337/dc16-2042 PMC6977405 27999003

[21] Ismail-Beigi F Craven T Banerji MA Basile J Calles J Cohen RM. Effect of Intensive Treatment of Hyperglycaemia on Microvascular Outcomes in Type 2 Diabetes: An Analysis of the Accord Randomised Trial. Lancet (2010) 376(9739):419–30. doi: 10.1016/S0140-6736(10)60576-4 PMC4123233 20594588

[22] Charles M Ejskjaer N Witte DR Borch-Johnsen K Lauritzen T Sandbaek A. Prevalence of Neuropathy and Peripheral Arterial Disease and the Impact of Treatment in People With Screen-Detected Type 2 Diabetes: The Addition-Denmark Study. Diabetes Care (2011) 34(10):2244–9. doi: 10.2337/dc11-0903 PMC3177734 21816977

[23] Duckworth W Abraira C Moritz T Reda D Emanuele N Reaven PD. Glucose Control and Vascular Complications in Veterans With Type 2 Diabetes. N Engl J Med (2009) 360(2):129–39. doi: 10.1056/NEJMoa0808431 19092145

[24] Recio-Pinto E Rechler MM Ishii DN. Effects of Insulin, Insulin-Like Growth Factor-Ii, and Nerve Growth Factor on Neurite Formation and Survival in Cultured Sympathetic and Sensory Neurons. J Neurosci (1986) 6(5):1211–9. doi: 10.1523/JNEUROSCI.06-05-01211.1986 PMC6568556 3519887

[25] Brussee V Cunningham FA Zochodne DW. Direct Insulin Signaling of Neurons Reverses Diabetic Neuropathy. Diabetes (2004) 53(7):1824–30. doi: 10.2337/diabetes.53.7.1824 15220207

[26] Zheng H Sun W Zhang Q Zhang Y Ji L Liu X. Proinflammatory Cytokines Predict the Incidence of Diabetic Peripheral Neuropathy Over 5 Years in Chinese Type 2 Diabetes Patients: A Prospective Cohort Study. EClinicalMedicine (2021) 31:100649. doi: 10.1016/j.eclinm.2020.100649 33385123 PMC7772538

[27] Papadopoulou-Marketou N Whiss PA Eriksson AC Hyllienmark L Papassotiriou I Wahlberg J. Plasma Levels of Tissue Inhibitor of Metalloproteinase-1 in Patients With Type 1 Diabetes Mellitus Associate With Early Diabetic Neuropathy and Nephropathy. Diabetes Vasc Dis Res (2021) 18(2):14791641211002470. doi: 10.1177/14791641211002470 PMC8481743 33775157

