# Supplementary material for: Association Between Fat Mass or Fat Fibrotic Gene Expression and Polyneuropathy in Subjects With Obesity: A Korean Metabolic Bariatric Surgery Cohort
Source: Front Endocrinol (Lausanne). 2022 May 16;13:881093. doi: 10.3389/fendo.2022.881093 (PMC9149169; doi:10.3389/fendo.2022.881093)
Supplement: Supplementary file 1 [file DataSheet_1.docx]

| **Table 1** **List of primer sequences** | | |
| --- | --- | --- |
| Gene | Primer probe | Sequence |
| *FN1* | Forward | 5'-TTCTAAGATTTGGTTTGGGATCAAT-3' |
|  | Reverse | 5’-TCTTGGTTGGCTGCATATGC-3’ |
| *TIMP1* | Forward | 5'-ACTTCCACAGGTCCCACAAC-3' |
|  | Reverse | 5'-TTTGCAGGGGATGGATAAAC-3' |
| *CCL2* | Forward | 5'-TCTGTGCCTGCTGCTCATAG-3' |
|  | Reverse | 5'-CAGATCTCCTTGGCCACAAT-3' |
| *CXCL8* | Forward | 5′-ATGACTTCCAAGCTGGCCGTGGCT-3′ |
|  | Reverse | 5′-TCTCAGCCCTCTTCAAAAACTTCT-3′ |
| *Cyclophilin* | Forward | 5′-TCTGCACTGCCAAGACTGAG-3′ |
|  | Reverse | 5′-TCGAGTTGTCCACAGTCAGC-3′ |
|  | | |
